# Chia Seeds (*Salvia hispanica* L.): Can They Be Used as Ingredients in Making Sports Energy Gel?

**DOI:** 10.3390/gels7040267

**Published:** 2021-12-16

**Authors:** Yanesti Nuravianda Lestari, Eko Farida, Nur Amin, Wiwik Afridah, Fifi Khoirul Fitriyah, Sunanto Sunanto

**Affiliations:** 1 Department of Nutrition, Faculty of Sport Science, Universitas Negeri Semarang, Semarang 50229, Indonesia; e_farida@mail.unnes.ac.id; 2 Department of Sport Science, Faculty of Health Science, Universitas Ngudi Waluyo, Semarang 50513, Indonesia; nuramin@unw.ac.id; 3 Department of Public Health, Faculty of Health Science, Universitas Nahdlatul Ulama Surabaya, Surabaya 60237, Indonesia; wiwik@unusa.ac.id; 4 Department of Early Childhood Education, Faculty of Teacher Training and Education, Universitas Nahdlatul Ulama Surabaya, Surabaya 60237, Indonesia; fifi@unusa.ac.id (F.K.F.); alif30@unusa.ac.id (S.S.)

**Keywords:** hydrocolloids, physicochemical, energy, mineral content, athletes, performance

## Abstract

Dehydration during exercise has been shown to limit performance. This study aimed to determine the best hydrocolloid for producing sports energy gel from chia seeds (*Salvia hispanica* L.). This study was a completed random-design study using one factor: the addition of 0.1% *w/w* hydrocolloids (SEG1: xanthan gum; SEG2: pectin; and SEG3: carboxymethyl cellulose). A sports energy gel was then analyzed for pH, viscosity, total soluble solids, potassium content, and gross energy. The sensory characteristics that were analyzed include color, texture, aroma, and flavor, using hedonic tests on 25 panelists. The addition of different hydrocolloids resulted in significant differences in pH, viscosity, total soluble solids, potassium, and energy contents (*p* = 0.026; 0.0001; 0.0001; and 0.0001). Differences in hydrocolloid types also led to differences in the panelists’ perceptions of the sports energy gels’ colors and textures (*p* = 0.008 and 0.0001). The best formulation was the sports energy gel with added xanthan gum, which showed the highest average energy, total soluble solids, potassium, and viscosity values, and the lowest average of pH values (60.24 ± 0.340, 10.6 ± 0.08, 19.6 ± 0.23, 367.4 ± 9.81, and 5.2 ± 0.38, respectively). The conclusion is that chia seeds can be used as the main ingredient for producing a high-energy sports gel. Energy has a huge impact on a person’s physical and mental health.

## 1. Introduction

Sports drinks are beverages designed to help athletes replenish their energy, nutrient, fluid, and electrolyte levels after exercise. Sports drinks are usually rich in carbohydrates, an efficient source of energy. Sports drinks also serve to maintain body balance, improve performance, and contain several electrolytes that can improve performance when consumed before or during exercise with a certain intensity [1,2].

The formulation of sports drinks should refer to the fulfillment of athletes’ specific energy, nutrient, fluid, and electrolyte needs, based on the exercise phase (before, during, or after exercise). One of the food ingredients used as a source of energy and nutrients in formulating sports drinks is chia seeds (*Salvia hispanica* L.). Chia seeds have a slightly ovular shape, are usually black, gray, or brown with tiny white spots. In water, chia seeds can expand and produce a clear white mucus. Chia seeds are often also used as components in the manufacturing of certain food products and serve as thickeners, emulsifiers, or stabilizers [3,4,5]. Chia seeds have high fiber and protein contents, are gluten-free, high in omega-3 fatty acids, rich in vitamins and minerals, and contain antioxidants (polyphenols, tocopherols, and isoflavones) [6,7]. The addition of chia seeds as a component in sports drinks can increase energy content while lowering the amount of fluid in sports drinks, as athletes are expected to consume high-calorie drinks with few fluids. Athletes who perform high-intensity endurance exercises are restricted from consuming excessive fluids during exercise, since they can cause discomfort to the digestive system [8,9].

On the other hand, athletes require adequate energy, nutrients, and electrolytes to support their performance. However, producing sports drinks that are dense in energy, nutrients, and electrolytes, but with few fluids, requires a component that can bind water and form a colloid called a hydrocolloid. Hydrocolloid components have several other uses, for example, as thickeners, gel-forming ingredients, emulsifiers, stabilizers, and coatings. The hydrocolloid components include carrageenan, cellulose derivate (carboxymethyl cellulose (CMC)), gelatin, chitosan, gum, pectin, and starch [10]. CMC is an anion derived from cellulose, consisting of carboxymethyl groups bound to the hydroxyl group of glucopyranose monomers and water-soluble polymers that can form a high viscosity in solutions [11]. A study stated that CMC’s addition as a hydrocolloid by 0.5–2% *w/w* can significantly increase chocolate beverages’ viscosity [12]. Another hydrocolloid component that is often used is pectin. Pectin has water-soluble characteristics but cannot be soluble in most organic solvents. Pectin-added foodstuffs have low viscosity but can form stable emulsions. Pectin’s addition, with a content of 0.5–1.5% *w/w*, can form products with a gel consistency. The addition of only 0.1% *w/w* of pectin is proven to maintain pH-stability during the shelf life of gel beverage products (12 days), and provide better rheological characteristics (viscosity) than the control [13,14]. Xanthan gum, a hydrocolloid gum group, is a type of hydrocolloid produced by the bacterial microorganism Xanthomonas campest, an extracellular anion polysaccharides with a molecular weight of 2.5 × 10^6^ g/mol [11]. The addition of hydrocolloid xanthan gum increases the product’s viscosity, shows consistency stability, and prevents syneresis during the storage of strawberry desserts [15]. In hydrocolloid components, athletes’ sports drinks are no longer liquid but in gel form.

This study aimed to determine the best hydrocolloids to produce sports energy gel from chia seeds (*Salvia hispanica* L.). This study targeted developing sports energy gel products that utilize chia seeds (*Salvia hispanica* L.) as raw materials and that are formulated using several hydrocolloids, such as xanthan gum, pectin, and carboxymethyl cellulose (CMC). Such products can be beneficial for athletes, serving as a high-energy drink that quickly boosts an athlete’s energy during exercise. This study was based on previous research that also conducted product development. Nevertheless, the resulting product was a sport drink based on chia seeds or energy drinks with hydrocolloid differences which were then analyzed for physical properties, chemistry, nutritional content, and sensory characteristics [1,13,16]. This research combined previous research in the hope of being able to produce better final product characteristics. This research used, as a basis for implementing the next stage of research, product trials with athletes, and the product was studied for its effect on the athlete’s endurance.

## 2. Results and Discussion

### 2.1. Characteristics of Physicochemical

#### 2.1.1. pH

Based on the results, the pH value of the sports energy gels was significantly different in all formulations, showed by *p*-values < 0.05 (*p* = 0.026). The highest pH value of the sports energy gels was the one with added CMC. Further test results, with Duncan’s multiple range tests, showed that the sports energy gel with added xanthan gum had a significantly different pH value than the sports energy gel with added pectin and CMC, while sports energy gel with added pectin and CMC had no significant pH value differences (Table 1).

The results showed that the pH of sports energy gel with hydrocolloid treatment produced a pH value that tended to be acidic. This result followed a previous study that examined the effect of adding different types and concentrations of hydrocolloids to mango filling, stating that hydrocolloids will decrease the pH value. The increase in the amounts of hydrocolloids added will further decrease the pH value of mango filling. However, the addition of different types of hydrocolloids did not result in significant differences in pH values. This result is due to the electrostatic interaction in the carbohydrate and protein components in the product’s composition, where the higher the concentration of hydrocolloids, the more hydrogen bonds are formed, so that the condition becomes acidic and will decrease water activity [17,18].

The study results stated a significant difference in pH value due to the addition of different hydrocolloids, which is supported by prior research that examined a chocolate beverage’s sensory and physical characteristics. The study stated that the pH value of chocolate drinks with added hydrocolloids (iota-carrageenan, kappa-carrageenan, and xanthan gum) showed significant differences [19]. It was known that the addition of xanthan gum showed the smallest pH value and was significantly different from the sport energy gel with added pectin and CMC. Xanthan gum is a hydrocolloid produced by substrate fermentation, by microorganisms of the genus Xanthomonas, strain Xanthomonas campestris NRRL B-1459. The fermentation results will produce products with a pH that tends to be acidic, so that if added to a foodstuff, it will affect the pH, which eventually becomes lower. In general, the proportion of the added gum and other types of hydrocolloids correlate directly to the acidity and pH of a product [20,21,22,23,24].

#### 2.1.2. Total Soluble Solids (TSS)

The results of the analysis stated that the total soluble solids in all three sports energy gel formulations were significantly different, showed by *p*-values < 0.05 (*p* = 0.0001). The highest percentage of the TSS was found in the sports energy gel with added xanthan gum. Based on further tests obtained, the sports energy gel with added pectin and CMC did not show a significantly different TSS percentage. However, the sports energy gel with added xanthan gum appeared to show a significantly different percentage of TSS when compared to sports energy gels with added pectin and CMC (Table 1).

The results showed that the TSS of the sports energy gels were significantly different due to different hydrocolloids. The study results were in line with previous research on the influence of hydrocolloid concentrations (agar) on the physicochemical and sensory characteristics of pumpkin leather, which stated that different agar concentrations caused significant differences in the total acid, total soluble solid, vitamin C, and water content [25]. The results of further tests showed that the total dissolved solids of sports energy gels with added pectin and CMC had no significant differences, although xanthan gum’s addition showed a marked difference in total dissolved solids. This result can also be seen in the texture of sports energy drinks with added CMC and pectin that were not-so-elastic, compared to sports energy drinks with added xanthan gum. This study’s results are in accordance with prior research that examined the influence of different types of hydrocolloids (agar, CMC, and Arabic gum) on the characteristics of papaya fruit leather, which stated that the addition of CMC and agar had no significant difference in total dissolved solids. However, both were significantly different for papaya fruit leather with added Arabic gum. Other studies also stated that adding hydrocolloids, in the form of CMCs, with variations in different concentrations is known to decrease the total soluble solids in bottle gourd juice. This condition can be attributed to the water content in the composition of sports energy gel, where the lower the water content, the higher the total soluble solids [26,27,28].

#### 2.1.3. Viscosity

It is known that viscosity in all three sports energy gel formulations showed significant differences, with a *p*-value < 0.05 (*p* = 0.0001). The viscosity of sports energy gel with added xanthan gum was significantly different from sports energy gels with added pectin and CMC. However, sports energy gels with added pectin did not show a significant difference in viscosity, compared to sports energy gels with added CMC (Table 1).

The results stated significant viscosity differences in sports energy gels, due to different hydrocolloids. This result was in line with the research on brown rice milk malt with different added stabilizing materials (CMC, kappa-carrageenan, pectin, and Na-alginate), which stated that the addition of different stabilizing materials had a noticeable effect on the viscosity of the product. Hydrocolloids have hydroxyl groups that can increase their affinity to bind and absorb water so that the water in the composition of the material becomes unable to move freely, resulting in increased viscosity. The difference in the final product’s viscosity can be influenced by the type and concentration of hydrocolloids and the pH and temperature of a product [29,30,31,32]. The number of hydroxyl groups present in a hydrocolloid can increase its affinity to bind water molecules or become more hydrophilic. Based on the results of the FTIR spectrum, xanthan gum consists of hydroxyl groups, carbonyl groups, carboxyl groups, and acetal groups. Other studies also state that commercial xanthan gum has 3386 hydroxyl groups, 1627 carbonyl groups, 1529 carboxyl groups, and 1160 acetal groups. Pectin is known to only have an active carbonyl group and a hydroxyl group in its molecular structure. Meanwhile, carboxymethyl cellulose (CMC) has a molecular structure consisting of a hydroxyl group and a carboxyl group. Xanthan gum, pectin, and CMC all have a hydroxyl group component in their molecular structure, which is associated with their ability to form a gel in a solution. However, the hydrocolloid ability to form a gel is not only influenced by the presence of hydroxyl groups in its molecular structure, but is also influenced by the temperature and pH of the solution. The sports energy gel with added xanthan gum showed the highest and most significant viscosity, compared to the sport energy gel with added pectin and CMC. This is attributed to the stability of xanthan gum over a wide temperature and pH range [10,33,34,35].

Further tests showed that sports energy gels with added pectin and CMC did not show significant viscosity differences. Viscosity is closely related to total soluble solids, where the results also stated that the sports energy gels with added pectin and CMC did not show significant differences in the total soluble solids. The sports energy gel with added xanthan gum showed a significant difference in viscosity compared to the sports energy gels with added pectin and CMC, where the viscosity value was the lowest. This result was associated with a low dose of xanthan gum addition (0.1% *w/w*). In line with the other studies, these results showed that products with added hydrocolloid CMC had higher viscosities than those with added sodium alginate, pectin, and gum acacia. In addition, other studies examining the effect of hydrocolloid addition on the viscosity and sensory characteristics of raspberry-juice milk showed that the addition of CMC improved the viscosity and stability of products, compared to products with added hydrocolloid pectin and kappa-carrageenan. Research on the addition of hydrocolloid type variations in the corn starch–rice flour system showed that the addition of hydrocolloids of any type increased the viscosity significantly [36,37,38].

#### 2.1.4. Potassium Content

The potassium content in sports energy gel was calculated for every 100 mL volume, where it is known that the potassium content in all three sports energy gel formulations is significantly different (*p* = 0.0001). The potassium content for each formulation of sports energy gel also showed a noticeable difference. The highest potassium content was found in the sports energy gel with added xanthan gum (Table 1).

The results stated significant differences in the potassium content of all three sports energy gel treatments. Further tests also stated that each sports energy gel with different added hydrocolloids showed significantly different potassium contents. This result was associated with the potassium in hydrocolloids, where each hydrocolloid had a different potassium content. The highest potassium content was shown in the sports energy gel with added xanthan gum. Xanthan gum is a hydrocolloid produced by *Xanthomonas xampestris* microorganisms with a high potassium content of 80 mg/100 g xanthan gum powder [29]. The potassium content in xanthan gum was higher than in CMC, where CMC, derived from cellulose, only had a potassium content of 19 mg/100 g. The mineral content in a hydrocolloid can be influenced by the essential ingredients of its production; one example is in the manufacturing of hydrocolloids using cocoa pod husks as a base material. It is known that the husks of cocoa pods are high in minerals, especially potassium and other minerals such as calcium, sodium, and magnesium [39,40].

#### 2.1.5. Energy Content

The energy content (gross energy) of all three sports energy gel formulations differs significantly, with a *p*-value < 0.05 (*p* = 0001). Each sports energy gel formulation also showed a significantly different energy content. The highest energy content was found in the sports energy gel with added xanthan gum (Table 1).

The study results stated significant differences in the energy contents of the three sports energy gel treatments, where each sports energy drink showed significant differences. This result can be attributed to the difference in the energy content of each type of hydrocolloid used. Based on the US Department of Agriculture’s central database, it is known that pectin in powder form has a total energy of 1360 kJ, equivalent to 325 kcal per 100 g of material. Xanthan gum had a much-higher total energy than pectin, of 625 kcal/100 g, while the total energy content in 100 g of CMC amounted to 320 kcal. The high energy in the final sports energy gel product was also due to the addition of chia seeds in the formulation, wherein 100 g of chia seeds contains 486 kcal of energy [7].

### 2.2. Sensory Characteristics

#### 2.2.1. Color

**Figure 1:**
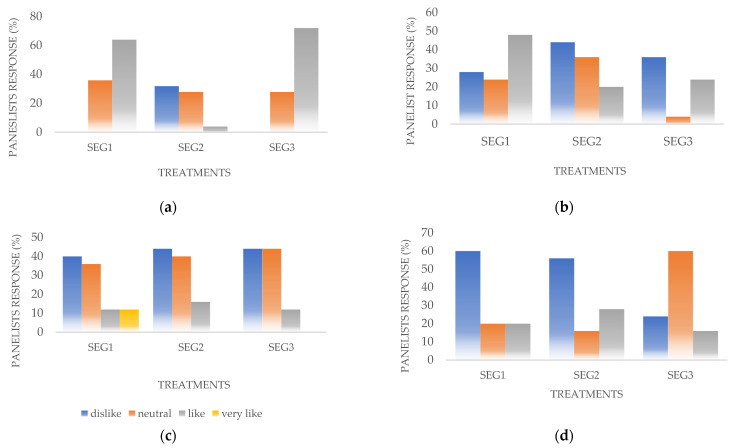
Panelist assessment on sensory characteristics. (**a**) The color of sports energy gel; (**b**) the texture of sports energy gel; (**c**) the aroma of sports energy gel; (**d**) the flavor of sports energy gel. The values are expressed as the percent of panelists (*n* = 25). Treatments: SEG1 = sports energy gel with the addition of xanthan gum; SEG2 = sports energy gel with pectin; and SEG3 = sports energy gel with CMC (carboxymethyl cellulose).

Hedonic test results showed that the panelist’s sports energy gel color preference was highest in the sports energy gel treatment with added CMC. Most of the panelists liked the color of all of the sports energy gel treatments (Figure 1a).

Based on the obtained statistical tests, panelists’ preference for sports energy gel color was significantly different, indicated by the value of *p* < 0.05 (*p* = 0.008). Further Bonferroni tests showed that the sports energy gel with added pectin showed significant differences in the color preference of panelists, compared to those with added xanthan gum or CMC. The sports energy gel with added xanthan gum did not significantly differ, in terms of the panelists’ color preference, from the gel with added CMC (Table 2).

The study results mentioned a difference in the perception of the panelists’ preference for sports energy gel color with different types of added hydrocolloids. This study’s results aligned with research on low-sugar cucumber sorbet with added hydrocolloids (CMC, xanthan gum, pectin, and agar), which states that differences in the type and percentage of hydrocolloids produce significantly different panelist perceptions of the color characteristics [40].

The results of another study also support research on pineapple puree with added xanthan gum hydrocolloids, which stated that the addition of different concentrations of xanthan gum (0%; 0.2%; 0.3%; 0.4%; 0.5%; 0.6%) produced a significantly different perception of the panelist’s preference for color characteristics. Research on pineapple puree with added xanthan gum also stated that, at xanthan gum concentrations of 0.2%, the best properties were shown [41].

#### 2.2.2. Texture

Based on the panelist’s assessment, the preferred sports energy gel texture was the gel with added xanthan gum. Most panelists did not like the texture of sports energy gels produced by adding pectin and CMC (Figure 1b). Statistical test results stated a significant difference in panelists’ preference of sports energy gel texture due to the addition of different types of hydrocolloids. This result is indicated by the *p*-value < 0.05 (*p* = 0.0001). Further, the Bonferroni tests showed that the panelists’ preference for the texture of the sports energy gel with added pectin was significantly different from the sports energy gel with added CMC. The addition of xanthan gum to the sports energy gel did not show a significant difference in the panelists’ level of preference, compared to the sports energy gels with added pectin and CMC (Table 2).

The study results mentioned differences between the panelists’ perceptions of the texture characteristics of sports energy gels with different added hydrocolloids. These results were supported by other studies on mango dates, which showed significant differences in texture hedonic values, due to different hydrocolloids (CMC and pectin) [42]. Another study on low-sugar cucumber sorbets also showed that the addition of different polysaccharides (CMC, xanthan gum, pectin, and agar) resulted in panelist assessments of significantly different texture characteristics [40]. Further test results stated that sports energy gels with added pectin showed noticeable differences in hedonic texture values when compared to sports energy gels with added CMC.

#### 2.2.3. Aroma

The statistical test results stated no difference in the panelist’s aroma preference for all hydrocolloid-addition treatments (*p* = 0.711) (Table 2). A hedonic test of aroma characteristics showed that most panelists disliked all of the sports energy gels’ aromas, although some panelists liked the aroma of the sports energy gel with added xanthan gum (Figure 1c).

The panelist’s assessment of the level of preference for the aromas of the sports energy gel with the addition of different hydrocolloid types did not show any significant differences. These results were in line with research that examined the influence of the addition of hydrocolloids (konjac; carrageenan; konjac and carrageenan) to the physical and organoleptic characteristics of papaya sheet jam, which showed that the addition of different types of hydrocolloids indicated no significant difference in the color of papaya sheet jam [43]. At the same time, most panelists stated that the three formulations of sports energy gel had a distinctive cucumber fragrance and savory aroma from mixing; other ingredients were similar. The aroma most preferred by panelists was in the sports energy gel with added xanthan gum, while the one with the lowest value of aroma preference was in the sports energy gel with added CMC.

These results were in line with the results of research conducted on low-sugar cucumber sorbets, where the addition of xanthan gum showed the highest cucumber aroma compared to other types of polysaccharides (CMC, pectin, and agar). The addition of pectin and CMC indicated the distinctive aroma of cucumbers in the order of second and third treatments [40].

#### 2.2.4. Flavor

Based on the panelist’s preference of flavor characteristics, it was obtained that most panelists did not like the flavor of the sports energy gel with added xanthan gum or pectin, but panelists still slightly liked the sports energy gel with added CMC (Figure 1d). The statistical test results showed no difference in the level of the panelists’ preference for the flavors of the sports energy gels (0.207) (Table 2).

The panelist’s assessment of their flavor preference for sports energy gels with the addition of hydrocolloids did not show any significant difference. These results were supported by research on the influence of types and concentrations of stabilizing substances on the quality of fruit leather mixed with guava and soursop, which stated that the addition of types and concentrations of stabilizing substances (CMC, gelatin, Arabic gum, and pectin) did not show significant flavor differences. This is in contrast to the research conducted on the physical and organoleptic properties of red fruit (*Pandanus conoideus* *Lamk*) puree, which showed that, in the formulation of products with the highest viscosity, the addition of CMC provided a taste intensity that was less-preferred by panelists [42,44].

### 2.3. The Best Formulation of Sports Energy Gel

Based on the De Garmo effectiveness index method’s results, the formulation with the highest value was SEG1 (Table 3). The sports energy gel with added xanthan gum showed the highest average energy, total soluble solids, potassium, and viscosity values, and the lowest average pH values. However, the average panelist’s preference for color, texture, aroma, and flavor characteristics did not indicate the highest value (Table 1 and Table 2).

Referring to the Codex Alimentarius Standard, beverage products can be low in energy if they contain 80 kJ per 100 mL, or equivalent to 20 kcal/100 mL [45]. Under South Africa’s claims regulation, beverage products can be claimed as high-energy if they contain 250 kJ/100 mL or equivalent to 59.75 kcal/100 mL [46]. Based on these provisions, the sports energy gel products in this study, particularly the best treatment product (SEG1), can be claimed as high energy sports drinks because they have an energy content of >59.75 kcal/100 mL (60.24 ± 0.340).

## 3. Conclusions

Based on the study results, it can be concluded that adding different types of hydrocolloids (xanthan gum, pectin, and CMC) produce pH, viscosity, total soluble solids, and potassium levels, as well as significantly different energy contents. The difference of hydrocolloid additions also led to differences in the panelist’s assessment of color and texture characteristics. The energy gel with added xanthan gum showed the highest average energy content, potassium, total soluble solids, and viscosity, and the lowest average pH value.

The limitation of this study is that the comparisons were only carried out in the treatment group without a control group (sports energy gel without the addition of hydrocolloids), so there was no baseline data of the physicochemical characteristics for comparison. This study result can be used as necessary information for further research on sports food formulations suitable for athletes. Further research can then be tested on specific characteristics to assess product delivery effectiveness, to obtain appropriate products. Future research is essential for evaluating this product for energy drinks for early childhood and development.

## 4. Materials and Methods

This study was conducted from April–August 2020 and was located in several laboratories: the culinary and dietetic laboratories and food processing laboratories of the Department of Public Health Sciences, Semarang State University, and the food science laboratories of the Faculty of Agricultural Technology, Soegijapranata Catholic University, Semarang.

This research was conducted using an experimental method with a completed random design with one factor (the addition of 0.1% *w/w* hydrocolloids consisting of 3 levels, as follows: (1) SEG1: sports energy gel with the addition of xanthan gum; (2) SEG2: sports energy gel with the addition of pectin; and (3) SEG3: sports energy gel with the addition of CMC (carboxymethyl cellulose). Each level of treatment was replicated three times for a total of nine treatments [13,16].

### 4.1. Sports Energy Gel Production

The ingredients used in the production of sports energy gel included chia seeds (*Salvia hispanica* L.), maltodextrin, cucumber (*Cucumis sativus*) juice, and dragon fruit (*Hylocereus polyrhizus*) juice, with added hydrocolloids (xanthan gum, pectin, and CMC). The ingredients were formulated into sports energy gels with compositions according to Table 4.

**Figure 2:**
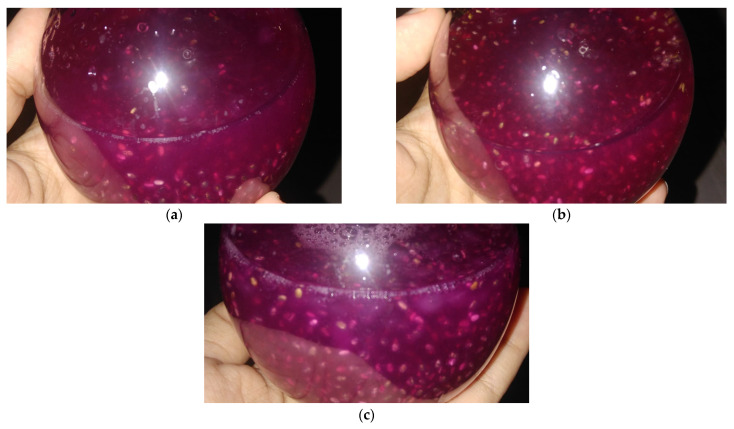
Chia seed-based sports energy gel with various hydrocolloids. (**a**) SEG1; (**b**) SEG2; (**c**) SEG3.

Treatments: SEG1 = sports energy gel with the addition of xanthan gum; SEG2 = sports energy gel with the addition of pectin; and SEG3 = sports energy gel with the addition of CMC (carboxymethyl cellulose) (Figure 2).

A sports energy gel is produced by mixing all ingredients, including hydrocolloids, into 200 mL of water, and then homogenated and heated over low heat for 10 min, until its color turns slightly darker [8].

### 4.2. Physicochemical Characteristics Analysis

The analyzed physical characteristics include pH, viscosity, total soluble solids, gross energy, and potassium levels. Gross energy analysis was conducted in the chemistry laboratory of the Faculty of Mathematics and Natural Sciences of Semarang State University. The pH, viscosity, total soluble solids, and potassium levels were analyzed in the Food Science Processing Faculty of Agricultural Technology, Soegiijapranata Catholic University of Semarang. Each sample of the study was analyzed three times for replication.

### 4.3. Sensory Characteristics Analysis

The sensory test conducted in this study used a hedonic test involving 25 semi-trained panelists. The tested sensory properties include appearance (color and consistency), aroma, flavor (level of sweetness and acidity), and texture in the mouth (viscosity). The panelist’s favorability for sensory characteristics was assessed using a 5-point Likert scale (1 = very dislike and 5 = very like), which was then transformed into an increasing numerical scale of 1–5 [13,47]

The characteristics of the subjects who were selected as panelists included: (1) healthy, not colorblind, and do not consume alcohol; (2) not smoking an hour before the implementation of the sensory test; (3) have no allergies or abstinence to one or more components of the foodstuffs to be sensory tested; (4) have experience carrying out sensory tests [48].

### 4.4. Determination of the Best Treatment

The determination of the best treatment of sports energy gel was made using the De Garmo effectiveness index method. The method was used to determine the treatment of hydrocolloids that produce the highest or the best value for all parameters of the analyzed sports energy gel (physical and sensory characteristics). The determination of the best treatment using this method was conducted in several stages [17,49]:

1. Panelists who had assessed the sports energy gel characteristics’ quality were asked to rank nine parameters of the sports energy gels: pH, total soluble solids, viscosity, potassium, gross energy, color, aroma, texture, and flavor. The results of the rankings were then summed and calculated on average for each parameter. The first rank parameter was the highest average value;
2. The BV (valence weight) was calculated for each parameter. BV is the level of importance for each analyzed parameter. BV values range from 0–1. The BV calculation used the following formula:
  (1) $$\mathrm{BV}\;=\frac{\mathrm{the}\;\mathrm{average}\;\mathrm{for}\;\mathrm{each}\;\mathrm{parameters}}{\mathrm{the}\;\mathrm{highest}\;\mathrm{value}\;\mathrm{of}\;\mathrm{parameters}}$$
3. The best and the worst values for each analyzed parameter was determined. The best and the worst values were the averages of each treatment for each parameter. If the best value is the maximum value, then the worst value is the minimum value, and vice versa. After that, the difference between the two values was calculated with the following formula:
  Δ = The best value − The worst value(2)
4. The BN (relative weight) was determined for each analyzed parameter. BN is the relative weight of each parameter analyzed. BN values come from BV, which is calculated using the following formula:
  (3) $${\mathrm{BN}}_{i}=\frac{{\mathrm{BV}}_{i}}{\sum_{i\;=1}^{p}{\mathrm{BV}}_{i}}\;;\;\overset{p}{\underset{i\;=1}{\sum }}{\mathrm{BN}}_{i}=1$$
  p = treatment.
5. The NE (effectiveness value) of each analyzed parameter was determined with the following formula:
  BV = (treatment value − the worst value)/Δ(4)
6. The NH (result value), which is the weight of the best treatment comparison against other parameters, was determined, calculated with the following formula:
  NH = BN × NE(5)
7. The following formula was used to calculate the effectiveness index of each treatment:
  (6) $$\mathrm{IE}\;=\overset{p}{\underset{i\;=1}{\sum }}{\mathrm{NH}}_{\mathrm{ij}}$$
  p = treatment.

### 4.5. Data Analysis

Physical properties data were analyzed using ANOVA one-way analysis, followed by a Duncan’s multiple range test (DMRT), with a confidence level of 95%, to determine the mean difference. The sensory properties data were analyzed using the Kruskal–Wallis test, followed by a Bonferroni comparison test (95% confidence level), to determine the difference between the treatments. The analysis was then continued to determine the best treatment level with the De Garmo effectiveness index method [19].

## Figures and Tables

**Table 1:** Physicochemical characteristics of chia seeds sports energy gel.

Treatment ^1^	Physicochemical Characteristics				
	Viscosity (cP)	pH	Total Solids (%)	Potassium (mg/100 mL)	Energy (Kcal/100 mL)
SEG1 *	367.4 ± 9.81 ^a^	5.2 ± 0.38 ^a^	10.6 ± 0.08 ^b^	19.6 ± 0.23 ^c^	60.24 ± 0.340 ^a^
SEG2 *	405.4 ± 2.89 ^b^	5.5 ± 0.13 ^b^	9.4 ± 0.01 ^a^	16.9 ± 1.01 ^a^	58.01 ± 0.317 ^b^
SEG3 *	423.9 ± 3.46 ^b^	5.9 ± 0.32 ^b,c^	9.7 ± 0.01 ^a^	17.9 ± 0.09 ^b^	41.83 ± 1.467 ^c^

^1^ The values are expressed as means ± SD (*n* = 9). Different tests of a mean of the treatments, using ANOVA one-way analysis, were significant at 0.05 (*p* = 0.0001; 0.026; 0.0001; 0.0001); significance is characterized by the notation *. A post hoc test, using Duncan’s multiple range test (DMRT) (the differences are shown with superscript letter notations (^a,b,c^)), indicated the significant differences between each treatment. Treatments (*n* = 9): SEG1 = sports energy gel with the addition of xanthan gum; SEG2 = sports energy gel with the addition of pectin; and SEG3 = sports energy gel with the addition of CMC (carboxymethyl cellulose).

**Table 2:** Sensory characteristics of sports energy gel.

Treatment ^2^	Sensory Characteristics			
	Color *	Texture *	Aroma	Flavor
SEG1	3.6 ± 0.49 ^b,c^	3.2 ± 0.87 ^a,b^	2.9 ± 1.02	2.6 ± 0.82
SEG2	3.08 ± 0.86 ^a^	2.8 ± 0.78 ^a^	2.8 ± 0.74	2.8 ± 0.89
SEG3	3.7 ± 0.46 ^c^	3.9 ± 0.78 ^b,c^	2.7 ± 0.69	2.9 ± 0.64

^2^ The values are expressed as means ± SD (*n* = 25). Different tests of mean using the Kruskal–Wallis test, significant at 0.05 (*p* = 0.008; 0.0001; 0.711; dan 0.207); significance is characterized by the notation *. Post hoc test using the Bonferroni test; the differences in superscript letter notation (^a,b,c^) indicate significant differences between each treatment. Treatments: SEG1 = sports energy gel with the addition of xanthan gum; SEG2 = sports energy gel with the addition of pectin; and SEG3 = sports energy gel with the addition of CMC (carboxymethyl cellulose).

**Table 3:** Determination of the effectiveness index.

Variables	BV	BN	SEG1		SEG2		SEG3	
			NE	NH	NE	NH	NE	NH
Energy	0.48259	0.080	1.000	0.080	0.879	0.070	0.000	0.000
Viscosity	0.68657	0.114	1.000	0.114	0.188	0.021	0.000	0.000
pH	0.51741	0.086	1.000	0.086	0.609	0.052	0.000	0.000
Total Soluble Solids	0.46766	0.077	1.000	0.077	0.000	0.000	0.004	0.000
Potassium	0.83085	0.137	1.000	0.137	0.000	0.000	0.392	0.054
Aroma	0.47264	0.078	1.000	0.078	0.143	0.011	0.000	0.000
Flavor	0.78607	0.130	0.000	0.000	0.375	0.049	1.000	0.130
Texture	1	0.165	0.393	0.065	0.000	0.000	1.000	0.165
Color	0.801	0.133	0.875	0.116	0.000	0.000	1.000	0.133
Total	6.0	1.000		0.753 ^3^		0.204		0.482

^3^ Determination of the best formulation using the De Garmo effectiveness index method, based on the rank choices of panelists (*n* = 25) for all parameters (energy, viscosity, pH, total soluble solids, potassium, sensory of aroma, flavor, texture, and color). BV = weight of valence; BN = relative weight; NE = effectiveness value; NH = result value. The best formulation is the highest score of NH; this is shown by the notation *. Treatments: SEG1 = sports energy gel with the addition of xanthan gum; SEG2 = sports energy gel with the addition of pectin; and SEG3 = sports energy gel with the addition of CMC (carboxymethyl cellulose).

**Table 4:** Composition of the sports energy gels [8].

Ingredients (% *w/w*)	Treatments ^4^		
	SEG1	SEG2	SEG3
Chia seeds	2	2	2
Maltodextrin	7.5	7.5	7.5
Dragon fruit juice	2.5	2.5	2.5
Cucumber juice	2.5	2.5	2.5
Xanthan gum	0.1	-	-
Pectin	-	0.1	-
CMC	-	-	0.1

^4^ Treatments (*n* = 9): SEG1 = sports energy gel with the addition of xanthan gum; SEG2 = sports energy gel with the addition of pectin; and SEG3 = sports energy gel with the addition of CMC (carboxymethyl cellulose).

## Data Availability

Data sharing not applicable.
